# Supplementary material for: Splice-Junction-Based Mapping of Alternative Isoforms in the Human Proteome
Source: Cell Rep. Author manuscript; Available in PMC 2020 Jan 15. (PMC6961840; doi:10.1016/j.celrep.2019.11.026)
Supplement: 2 [file NIHMS1546469-supplement-2.zip › DF1/PXD010154/Esophagus/Esophagus_34_RBX1_HIMDLCMGTPSGTNSGAGK.pdf]

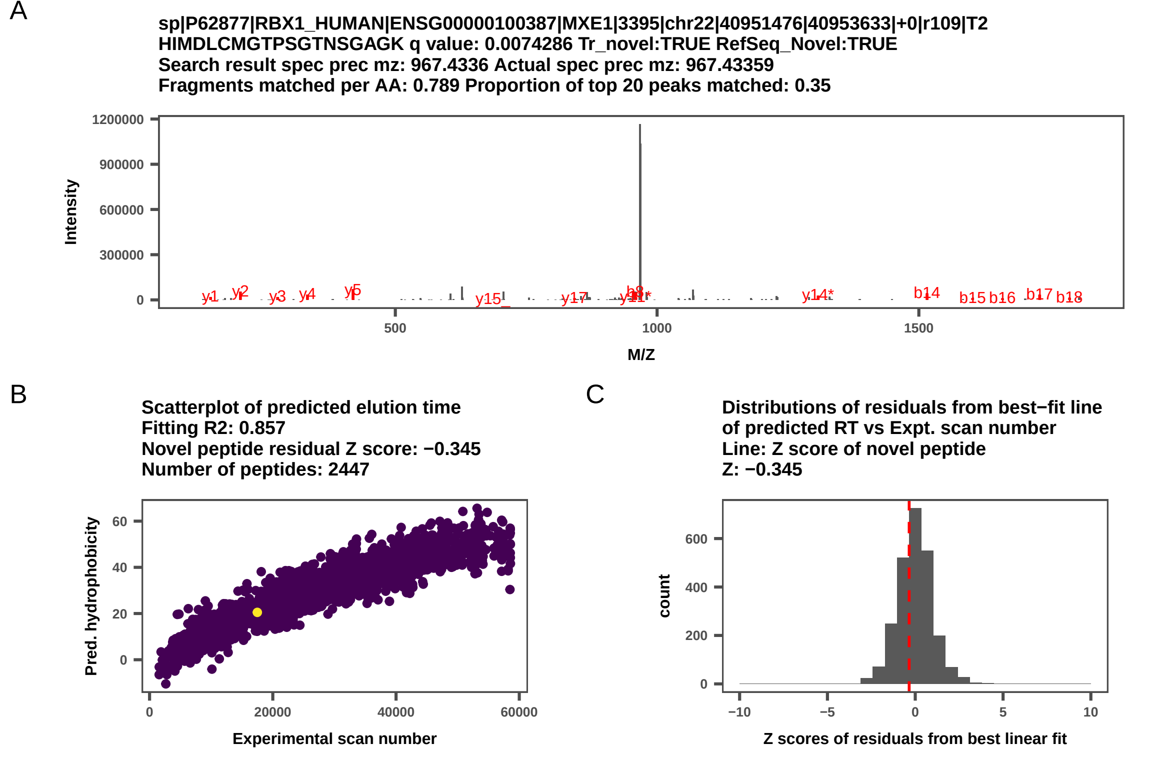
A
sp|P62877|RBX1_HUMAN|ENSG00000100387|MXE1|3395|chr22|40951476|40953633|+0|r109|T2
HIMDLCMGTPSGTNSGAGK q value: 0.0074286 Tr_novel:TRUE RefSeq_Novel:TRUE
Search result spec prec mz: 967.4336 Actual spec prec mz: 967.43359
Fragments matched per AA: 0.789 Proportion of top 20 peaks matched: 0.35
Intensity
1200000
900000
600000
300000
0
y1
y2
y3
y4
y5
y15_
y17
b8
y11*
y14*
b14
b15
b16
b17
b18
500
1000
1500
M/Z
B
Scatterplot of predicted elution time
Fitting R2: 0.857
Novel peptide residual Z score: −0.345
Number of peptides: 2447
Pred. hydrophobicity
60
40
20
0
0
20000
40000
60000
Experimental scan number
C
Distributions of residuals from best−fit line
of predicted RT vs Expt. scan number
Line: Z score of novel peptide
Z: −0.345
count
600
400
200
0
−10
−5
0
5
10
Z scores of residuals from best linear fit
